# Supplementary material for: Succinate utilisation by Salmonella is inhibited by multiple regulatory systems
Source: PLoS Genet. 2024 Mar 8;20(3):e1011142. doi: 10.1371/journal.pgen.1011142 (PMC10965054; doi:10.1371/journal.pgen.1011142)
Supplement: S1 Text — (DOCX) [file pgen.1011142.s001.docx]

**S1 Text:**

**Hfq, PNPase and their cognate sRNAs maintain the inhibition of succinate utilisation**

Our discovery that the inactivation of the RNA binding proteins Hfq and PNPase promoted *Salmonella* growth upon succinate (Fig 3) led us to investigate the phenotype in more detail. In liquid culture, the ∆*hfq* and ∆*pnp* mutants displayed a lag time of ~5 hours and ~15 hours, respectively (S5A Fig), prompting experiments to investigate the role of small regulatory RNAs (sRNAs) in the inhibition of succinate utilisation.

The RNA chaperone Hfq and its associated sRNAs are key post-transcriptional regulatory determinants [1,2]. In *E. coli*, the sRNAs RybB, RyhB (RyhB-1 in *Salmonella*) and Spot42 (Spf) base-pair with the *sdhC* 5’-UTR to repress *sdhC* translation in an Hfq-dependent manner. In addition, RybB and RyhB reduce the stability of the *sdh* mRNA [3,4]. We reasoned that the observed Succ^+^ phenotype of the Hfq null mutant could reflect de-repression of the *sdh* mRNA at the translational level.

In *E. coli*, the iron-dependent sRNA RyhB represses growth with succinate under iron-limited conditions [5]. Because exogenous iron was not added to our M9 media, we investigated whether the inhibition of *Salmonella* growth with succinate was the consequence of the *sdh* repression by RyhB-1 or RyhB-2, the RyhB-1 paralog in *Salmonella* [6]. Neither iron (FeCl_3_) supplementation (up to 100 µM) or the double inactivation of RyhB-1 and RyhB-2 (strain ∆*ryhB-1/2*) generated a Succ^+^ phenotype (S6 Fig). Similarly, the simultaneous inactivation of four sRNAs (RybB, Spf, RyhB-1 and RyhB-2) did not affect growth on M9+Succ (S5B Fig).

Hfq and sRNAs are crucial for the stimulation of *rpoS* translation. The long 5’-UTR of *rpoS* mRNA forms a self-inhibitory hairpin secondary structure, that blocks the ribosome access to the ribosome binding site, repressing *rpoS* mRNA translational initiation [7]. In *E. coli*, base-pairing of the sRNAs ArcZ, DsrA and RprA with the *rpoS* 5’-UTR, relieves this self-repression in an Hfq-dependent manner to stimulate *rpoS* translation [8]. In addition, binding of ArcZ, DsrA and RprA to the *rpoS* 5’-UTR prevents the premature Rho-dependent transcription termination of the *rpoS* mRNA [9]. As RpoS plays a pivotal role in the control of succinate utilisation, we assessed the growth of the triple ∆*arcZ* *rprA* *dsrA* mutant in M9+Succ. In comparison with the WT strain, no obvious differences were observed (S5B Fig). However, the successive deletion of sRNAs *arcZ*, *dsrA* and *rprA* in the ∆*rybB* *spf* *ryhB-1/2* genetic background did promote growth on succinate, and gradually reduced the duration of lag time (S5C Fig).

Inactivation of *pnp* is known to restore growth of a RyhB-overexpressing *E. coli* strain on succinate by reducing the stability of several sRNAs, including RyhB [10]. The same study demonstrated that the translational activation of *rpoS* by RprA and DsrA was attenuated in the ∆*pnp* background. To test whether PNPase inactivation boosted succinate utilisation through RpoS attenuation, we assessed the growth of a ∆*pnp* mutant that overexpressed *rpoS*. The plasmid-borne overexpression of *rpoS* in this strain totally suppressed the Succ^+^ phenotype (S5D Fig), consistent with the stimulation of RpoS expression by PNPase.

Taken together, these results indicate that the fast growth of the ∆*hfq* and ∆*pnp* strains reflected both the dysregulation of the sRNA-mediated repression of *sdh* and the activation of *rpoS* translation. However, none of the sRNA mutants tested displayed the same fast-growing pattern of the ∆*hfq* mutant, suggesting that other sRNAs may be involved in the inhibition of succinate utilisation.

**References:**

1. Santiago-Frangos A, Woodson SA. Hfq chaperone brings speed dating to bacterial sRNA. Wiley Interdiscip Rev RNA. 2018. doi:10.1002/wrna.1475

2. Vogel J, Luisi BF. Hfq and its constellation of RNA. Nature Reviews Microbiology. 2011. doi:10.1038/nrmicro2615

3. Massé E, Escorcia FE, Gottesman S. Coupled degradation of a small regulatory RNA and its mRNA targets in *Escherichia coli*. Genes Dev. 2003. doi:10.1101/gad.1127103

4. Desnoyers G, Massé E. Noncanonical repression of translation initiation through small RNA recruitment of the RNA chaperone Hfq. Genes Dev. 2012. doi:10.1101/gad.182493.111

5. Massé E, Gottesman S. A small RNA regulates the expression of genes involved in iron metabolism in *Escherichia coli*. Proc Natl Acad Sci U S A. 2002. doi:10.1073/pnas.032066599

6. Kim JN, Kwon YM. Genetic and phenotypic characterization of the RyhB regulon in *Salmonella* Typhimurium. Microbiol Res. 2013. doi:10.1016/j.micres.2012.06.007

7. Battesti A, Majdalani N, Gottesman S. The RpoS-Mediated General Stress Response in *Escherichia coli* . Annu Rev Microbiol. 2011. doi:10.1146/annurev-micro-090110-102946

8. Soper T, Mandin P, Majdalani N, Gottesman S, Woodson SA. Positive regulation by small RNAs and the role of Hfq. Proc Natl Acad Sci U S A. 2010. doi:10.1073/pnas.1004435107

9. Sedlyarova N, Shamovsky I, Bharati BK, Epshtein V, Chen J, Gottesman S, et al. sRNA-Mediated Control of Transcription Termination in *E. coli*. Cell. 2016. doi:10.1016/j.cell.2016.09.004

10. De Lay N, Gottesman S. Role of polynucleotide phosphorylase in sRNA function in *Escherichia coli*. RNA. 2011. doi:10.1261/rna.2531211
