# Supplementary material for: Succinate utilisation by Salmonella is inhibited by multiple regulatory systems
Source: PLoS Genet. 2024 Mar 8;20(3):e1011142. doi: 10.1371/journal.pgen.1011142 (PMC10965054; doi:10.1371/journal.pgen.1011142)
Supplement: S2 Text — (DOCX) [file pgen.1011142.s002.docx]

**S2 Text :**

**Anti-Shine-Dalgarno mutations and sub-inhibitory concentration of chloramphenicol boost succinate utilisation**

We identified a novel class of mutations that boost succinate utilisation by altering the anti-Shine-Dalgarno sequence (aSD) of the 16S ribosomal RNAs (rRNAs). Specifically, aSD SNPs in *rrsA* and *rrsH* genes that encode two of the seven 16S rRNAs present in *Salmonella* genomes [1] were found (alleles *rrsA^mut^* and *rrsH^mut^*, S11A Fig). Mature 16S rRNAs are assembled with ribosomal proteins to form the 30S ribosomal subunits that initiate mRNA translation [2,3]. Each 16S rRNA 3’-end carries an aSD motif (CCUCCUU) that base-pairs with the Shine-Dalgarno sequence (SD) on mRNA, promoting translational initiation at the start codon [4,5].

The SNPs carried by the *rrsA^mut^* and *rrsH^mut^* strains dramatically stimulated growth of *Salmonella* in M9+Succ, reducing the lag time to ~7 hours. When *E. coli* grows under nutrient limitation, the relative transcription of the *rrnH* rRNA operon increases and the resulting pool of RrsH-containing ribosomes can modulate the stress response by stimulating RpoS translation or stability [6]. Therefore, we reasoned that the aSD mutations may totally inactivate the rRNAs resulting in the reduction of RpoS expression. However, deletion of the *rrsA* and *rrsH* loci did not result in a Succ^+^ phenotype (S11C Fig). The plasmid-borne expression of *rpoS* only marginally increased the lag time of the *rrsA^mut^* and *rrsH^mut^* mutant strains, indicating that the mutations in the 16S rRNAs stimulate succinate utilisation, at least partially, in a RpoS-independent manner ( S11D and S11E Fig).

In *E. coli*, 16S rRNAs that carry a mutated aSD motif are processed and assembled into functional 30S subunits, which can initiate translation at the correct start codon [7]. This suggests that the mutated 16S rRNA RrsA^mut^ and RrsH^mut^ are assembled normally, and the presence of the resulting altered ribosome stimulates *Salmonella* growth upon succinate.

The aSD mutations prompted us to experiment with a translational inhibitor. We observed that subinhibitory concentrations of chloramphenicol (Cm) stimulated growth of 4/74 WT upon succinate (S11F Fig). The shortest lag time (~8 hours) was observed at a Cm concentration of 1.5 µg/mL. Addition of Cm caused a similar level of growth stimulation for *S.* Enteritidis strain P125109 (S12A Fig), indicating that the phenomenon is conserved in other *Salmonella* serovars.

Cm targets the 50S ribosome subunits to block translation [8]. Subinhibitory concentrations of this antibiotic prevent the RelA-mediated synthesis of the alarmone (p)ppGpp, the key signal molecule of the stringent response [9]. During amino acid starvation, (p)ppGpp accumulation is known to promote the transcription, translation and stability of RpoS [10], raising the possibility that the aSD mutations and Cm stimulate succinate utilisation directly through RpoS attenuation. Tetracycline (Tc) and other translation-inhibiting antibiotics also inhibit (p)ppGpp synthesis in *E. coli* [11], prompting us to test subinhibitory concentrations of Tc hydrochloride (1 and 2 µg/mL). However, Tc did not stimulate the growth of 4/74 at the concentrations tested (S12B Fig), suggesting that Cm does not reduce RpoS expression, *via* the inhibition of the stringent response.

Taken together, our findings suggest that the impairment of the ribosomal machinery by aSD mutations or by the presence of chloramphenicol impose a translational stress that stimulates genes involved in succinate utilisation. In line with this hypothesis, the inactivation of the translational elongation factor EF-P [12,13] also stimulated the growth of *Salmonella* upon succinate [14]. However, the link between protein biosynthesis impairment and the stimulation of succinate utilisation remains enigmatic. Further work will be required to decipher the regulatory mechanism that underpins this phenomenon.

**References:**

1. Lehner AF, Harvey S, Hill CW. Mapping and spacer identification of rRNA operons of *Salmonella typhimurium*. J Bacteriol. 1984. doi:10.1128/jb.160.2.682-686.1984

2. Laursen BS, Sørensen HP, Mortensen KK, Sperling-Petersen HU. Initiation of Protein Synthesis in Bacteria. Microbiol Mol Biol Rev. 2005. doi:10.1128/mmbr.69.1.101-123.2005

3. Gualerzi CO, Pon CL. Initiation of mRNA translation in bacteria: Structural and dynamic aspects. Cellular and Molecular Life Sciences. 2015. doi:10.1007/s00018-015-2010-3

4. Shine J, Dalgarno L. The 3’ terminal sequence of *Escherichia coli* 16S ribosomal RNA: complementarity to nonsense triplets and ribosome binding sites. Proc Natl Acad Sci U S A. 1974. doi:10.1073/pnas.71.4.1342

5. Steitz JA, Jakes K. How ribosomes select initiator regions in mRNA: base pair formation between the 3’ terminus of 16S rRNA and the mRNA during initiation of protein synthesis in *Escherichia coli*. Proc Natl Acad Sci U S A. 1975. doi:10.1073/pnas.72.12.4734

6. Kurylo CM, Parks MM, Juette MF, Zinshteyn B, Altman RB, Thibado JK, et al. Endogenous rRNA Sequence Variation Can Regulate Stress Response Gene Expression and Phenotype. Cell Rep. 2018. doi:10.1016/j.celrep.2018.08.093

7. Saito K, Green R, Buskirk AR. Translational initiation in *E. coli* occurs at the correct sites genome-wide in the absence of mRNA-rRNA base-pairing. Elife. 2020. doi:10.7554/eLife.55002

8. Wilson DN. Ribosome-targeting antibiotics and mechanisms of bacterial resistance. Nature Reviews Microbiology. 2014. doi:10.1038/nrmicro3155

9. Hobbs JK, Boraston AB. (p)ppGpp and the Stringent Response: An Emerging Threat to Antibiotic Therapy. ACS Infect Dis. 2019. doi:10.1021/acsinfecdis.9b00204

10. Battesti A, Majdalani N, Gottesman S. The RpoS-Mediated General Stress Response in *Escherichia coli* . Annu Rev Microbiol. 2011. doi:10.1146/annurev-micro-090110-102946

11. Kudrin P, Varik V, Oliveira SRA, Beljantseva J, Del Peso Santos T, Dzhygyr I, et al. Subinhibitory concentrations of bacteriostatic antibiotics induce relA-dependent and relA-independent tolerance to β-lactams. Antimicrob Agents Chemother. 2017. doi:10.1128/AAC.02173-16

12. Ude S, Lassak J, Starosta AL, Kraxenberger T, Wilson DN, Jung K. Translation elongation factor EF-P alleviates ribosome stalling at polyproline stretches. Science (80- ). 2013. doi:10.1126/science.1228985

13. Starosta AL, Lassak J, Jung K, Wilson DN. The bacterial translation stress response. FEMS Microbiology Reviews. 2014. doi:10.1111/1574-6976.12083

14. Hersch SJ, Radan B, Ilyas B, Lavoie P, Navarre WW. A stress-induced block in dicarboxylate uptake and utilization in *Salmonella*. J Bacteriol. 2021; JB.00487-20. doi:10.1128/JB.00487-20
