# Supplementary figures and images for: Succinate utilisation by Salmonella is inhibited by multiple regulatory systems
Source: PLoS Genet. 2024 Mar 8;20(3):e1011142. doi: 10.1371/journal.pgen.1011142 (PMC10965054; doi:10.1371/journal.pgen.1011142)

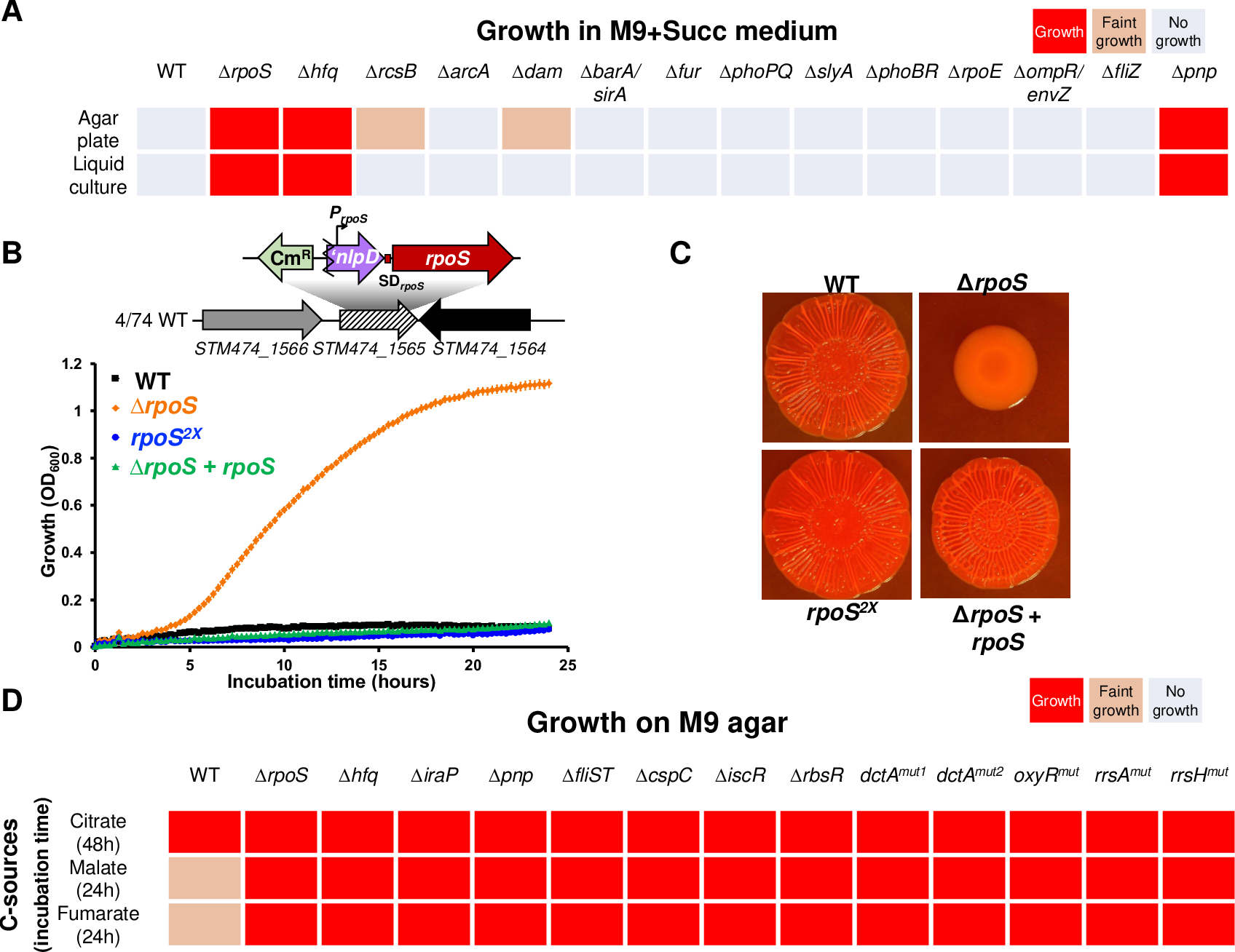

Supplement: S1 Fig — (A) the growth of a collection of S. Typhimurium 4/74 mutants lacking regulatory proteins was assessed on solidified M9+Succ agar and in liquid M9+Succ medium (in microplates), revealing the fast growth of mutants ΔrpoS (JH3674), Δhfq (JH3584) and Δpnp (JH3649). (B&C) Chromosomal complementation of the ΔrpoS mutation: a copy of rpoS (including its native promoter, bent arrow), linked to the cat Cm resistance gene was inserted in the non-transcribed pseudogene STM474_1565 of 4/74 WT (strain 4/74 rpoS2X, SNW226) and of ΔrpoS (ΔrpoS + rpoS, JH4160). The STM474_1565 gene is also known as STM1553 or SL1344_1483. RpoS-dependent phenotypes were assessed for each strain: growth was tested in M9+Succ (B) and RDAR phenotype was tested on Congo Red agar plates, confirming the Succ- RDAR+ of the complemented strain ΔrpoS + rpoS. (D) The growth of the novel Succ+ mutants identified (presented in Fig 3) was tested on solidified M9 minimal medium supplemented with 40 mM citrate, malate or fumarate. The growth was assessed with biological triplicates after the indicated incubation time (37°C) and the growth of each mutant was compared to 4/74 WT (Succ-) and ΔrpoS (Succ+). (TIF) [file pgen.1011142.s003.tif]

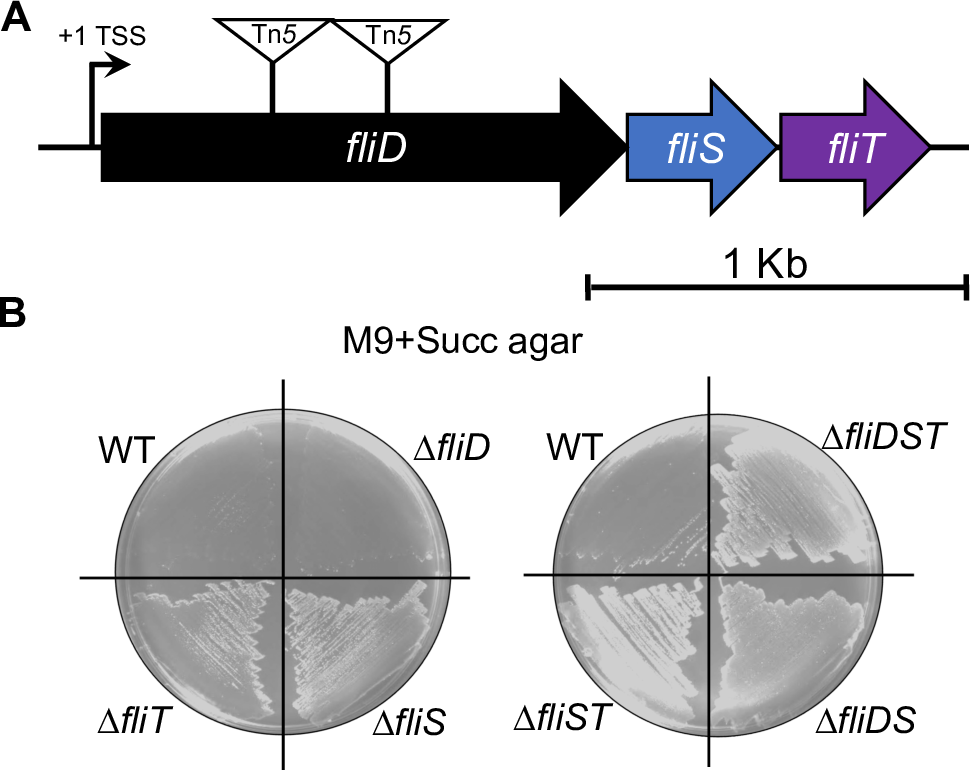

Supplement: S2 Fig — (A) Schematic representation of the fliDST operon. The transcription start site (+1 TSS) and the two Tn5 transposon insertions causing Succ+ phenotype are depicted (Table 1). (B) The inactivation of the flagellar chaperones FliS and FliT stimulates Salmonella growth with succinate. The growth of 4/74 WT and of mutants ΔfliD (SNW278), ΔfliS (SNW280), ΔfliT (SNW282), ΔfliDST (SNW284), ΔfliDS (SNW286), ΔfliST (SNW288) was assessed on M9+Succ agar plates after 48 hours of incubation at 37°C. (TIF) [file pgen.1011142.s004.tif]

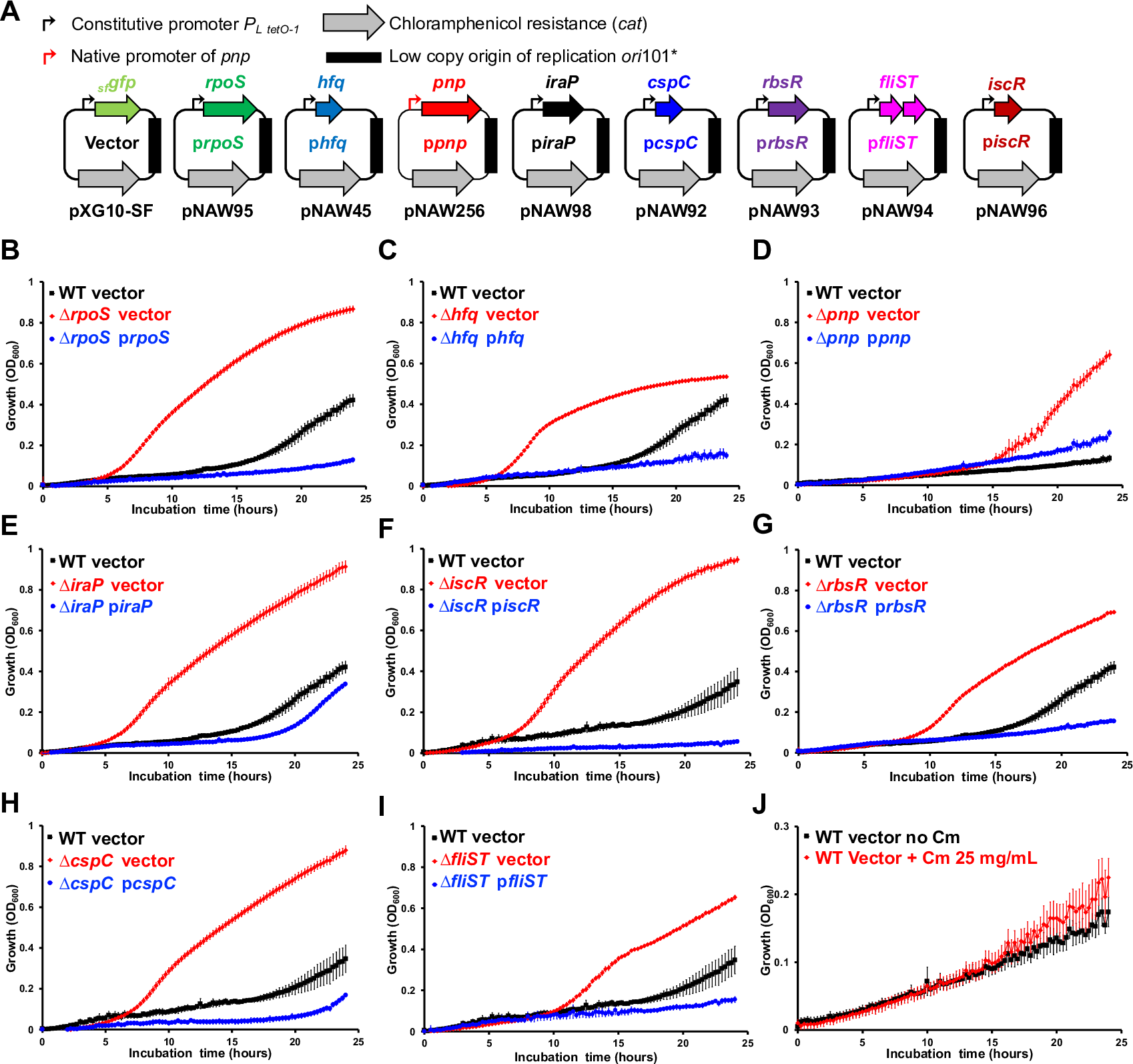

Supplement: S3 Fig — (A) The plasmids used for the complementation experiments are depicted and were constructed (Methods) using the backbone of pXG10-SF [99], a low-copy plasmid encoding for the cat resistance gene and carrying the ori101* replicon. Each plasmid carries the gene(s) of interest under the control of the strong constitutive promoter PL tetO-1 [101], except for pnp, that is controlled by its native promoter. For each growth curve (B-I), the plasmid pXG10-SF (“vector”) expressing the lacZ186::sfgfp fusion was used as a negative control. The strains 4/74 WT, ΔrpoS (JH3674), Δhfq (JH3584), Δpnp (JH3649), ΔiraP (SNW188), ΔiscR (SNW184), ΔrbsR (SNW294), ΔcspC (SNW292) and ΔfliST (SNW288) carrying the indicated plasmids were grown in M9+Succ, supplemented with 25 μg/mL Cm. (J) The presence of Cm (25 μg/mL) in M9+Succ stimulates mildly the growth of 4/74 WT carrying pXG10-SF. The growth curves were carried out with 6 replicates in 96-well plates. (TIF) [file pgen.1011142.s005.tif]

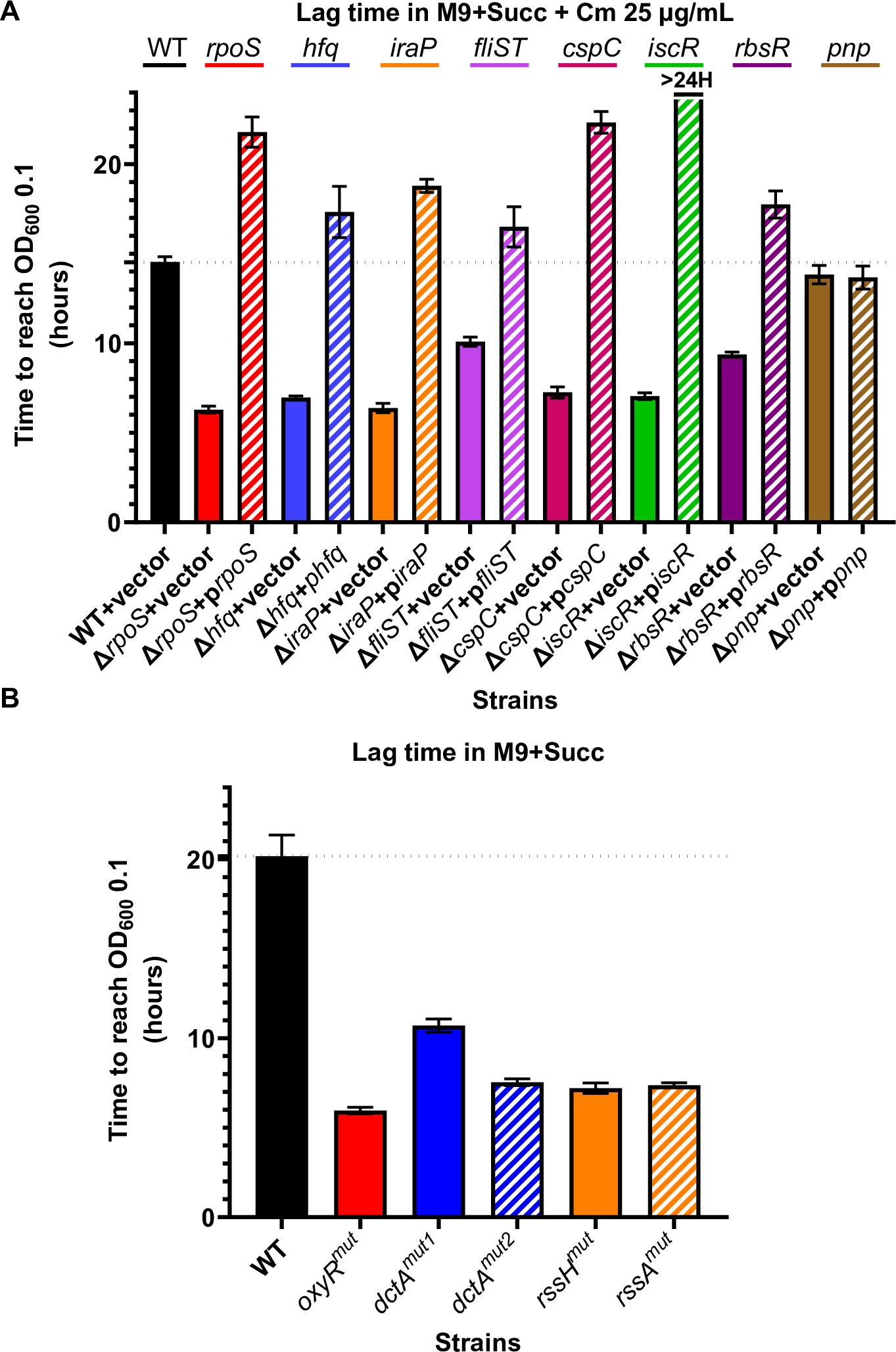

Supplement: S4 Fig — (A) The lag time was defined as the time required to reach an OD600 of 0.1. The lag time was determined for the regulatory mutants, ΔrpoS, Δhfq, ΔiraP, ΔfliST, ΔcspC, ΔiscR ΔrbsR, and Δpnp and for the corresponding complemented strains, based on the growth curves presented in S3 Fig. (B) The lag time was determined for the mutants oxyRmut, dctAmut1, dctAmut2, rssHmut and rrsAmut, based on the growth curves presented in Figs 5, 6, and S11. (TIF) [file pgen.1011142.s006.tif]

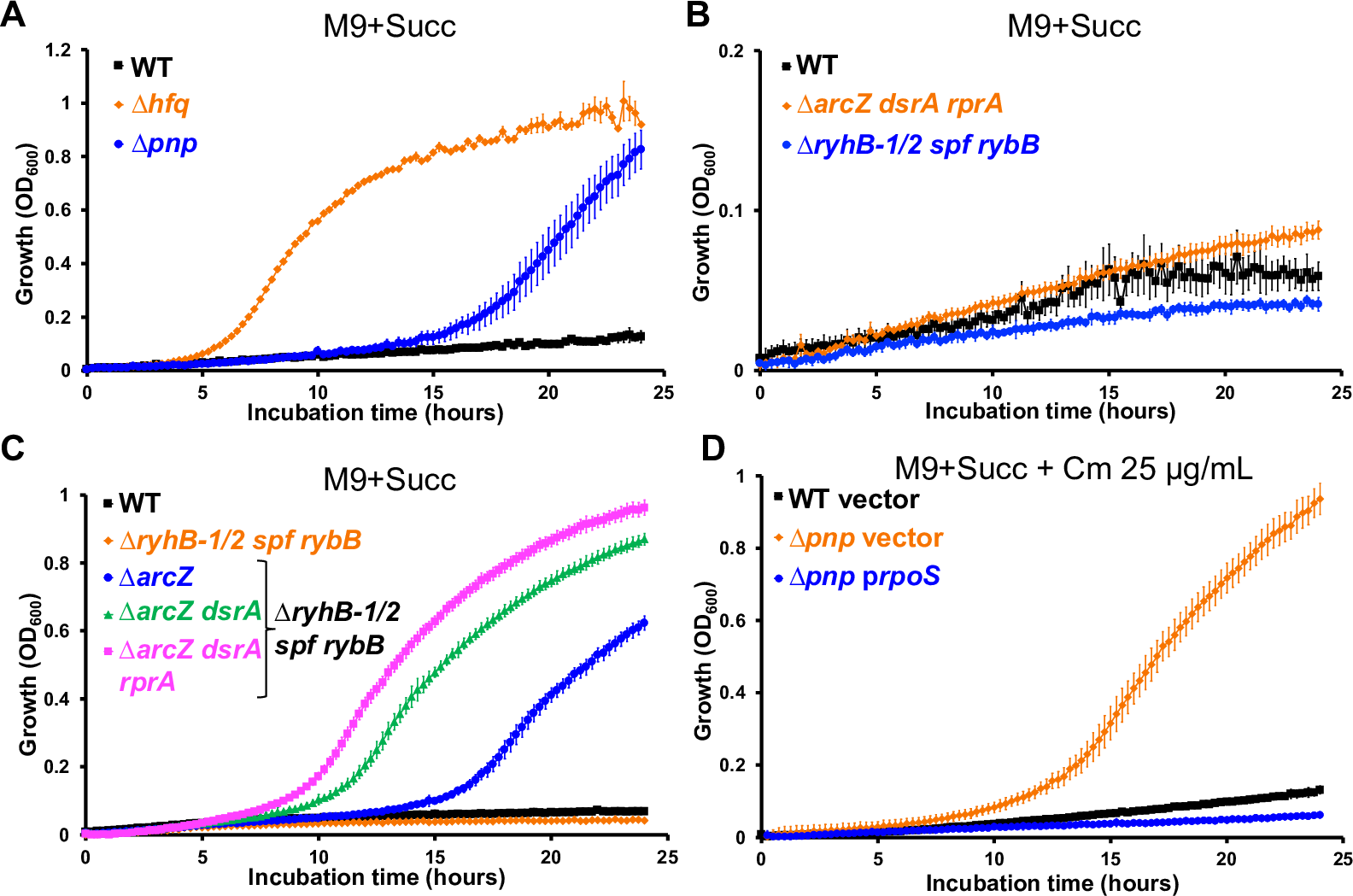

Supplement: S5 Fig — (A) Hfq and PNPase inactivation boosts Salmonella growth on succinate. (B) The co-inactivations of the rpoS activating sRNAs ArcZ, DsrA and RprA or of the sdh repressing sRNAs RyhB-1/2, Spf and RybB did not stimulate Salmonella growth with succinate. (C) Successive inactivations of ArcZ, DsrA and RprA in the ΔryhB-1 ryhB-2 rybB spf genetic background stimulate gradually the growth with succinate. (D) The overexpression of rpoS abolishes totally the Succ+ phenotype of the Δpnp mutant, lacking PNPase: growth was assessed for strains 4/74 WT and Δpnp, carrying the empty plasmid (vector, pNAW125) or the prpoS (pNAW95) plasmid, overexpressing rpoS. The strains used were all 4/74 derivatives: Δhfq (JH3584), Δpnp (JH3649), ΔarcZ dsrA rprA (JH4385), ΔryhB-1 ryhB-2 rybB spf (SNW630), ΔryhB-1 ryhB-2 rybB spf arcZ (SNW639), ΔryhB-1 ryhB-2 rybB spf arcZ dsrA (SNW640) and ΔryhB-1 ryhB-2 rybB spf arcZ dsrA rprA (SNW641). The medium used is indicated for each experiment. Growth curves were carried out with 6 replicates grown in 96-well plates, as specified in Methods. (TIF) [file pgen.1011142.s007.tif]

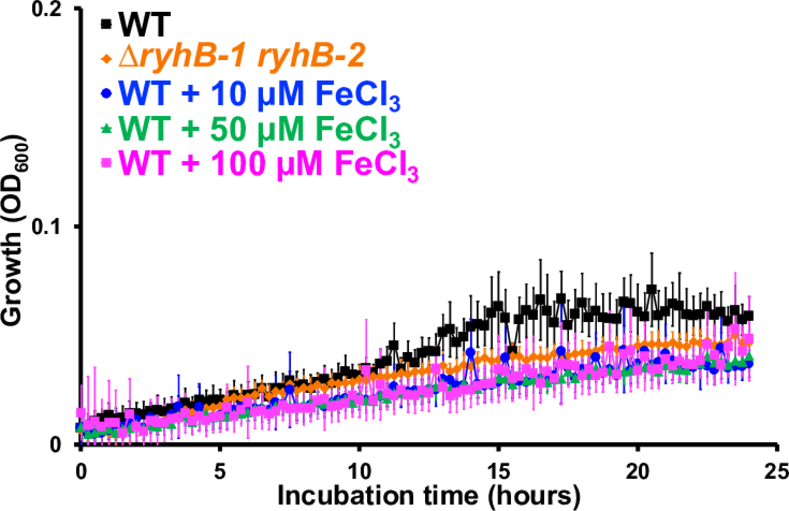

Supplement: S6 Fig — Strains 4/74 and ΔryhB-1 ryhB-2 (ΔryhB-1/2, JH4390) were grown in M9+Succ medium supplemented or not with iron (FeCl3) at the indicated concentration. The growth curves were carried out with 6 replicates in 96-well plates in the indicated medium. (TIF) [file pgen.1011142.s008.tif]

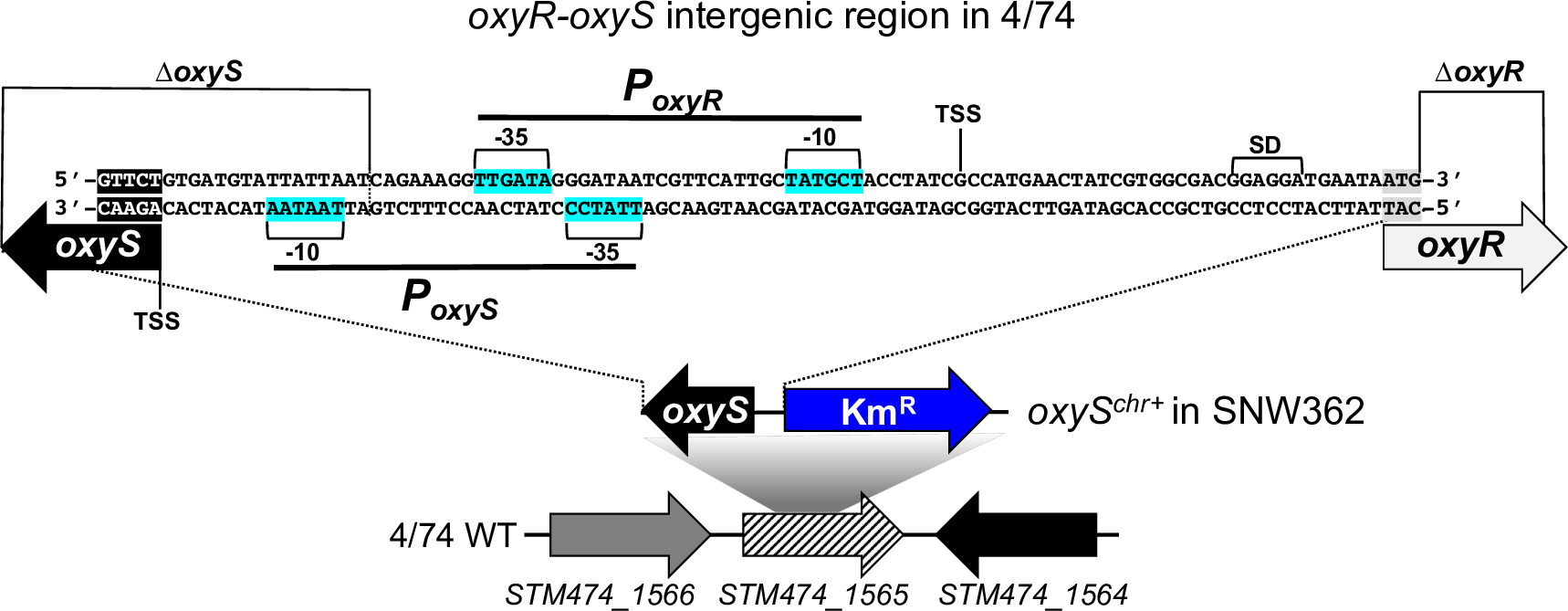

Supplement: S7 Fig — The intergenic region sequence is depicted and the -35 and -10 boxes of the PoxyS and PoxyR promoters are highlighted in blue, according to the corresponding locus of E. coli K-12 [52]. The ΔoxyS and ΔoxyR mutation are indicated. The transcription start sites (TSS) are indicated, according to the SalcomMac transcriptomic database [88,111]. For the complementation of the ΔoxyS mutation in strain SNW362 (oxySchr+), the oxyS gene, its native promoter and a KmR cassette were inserted into the non-transcribed pseudogene STM474_1565 [95]. (TIF) [file pgen.1011142.s009.tif]

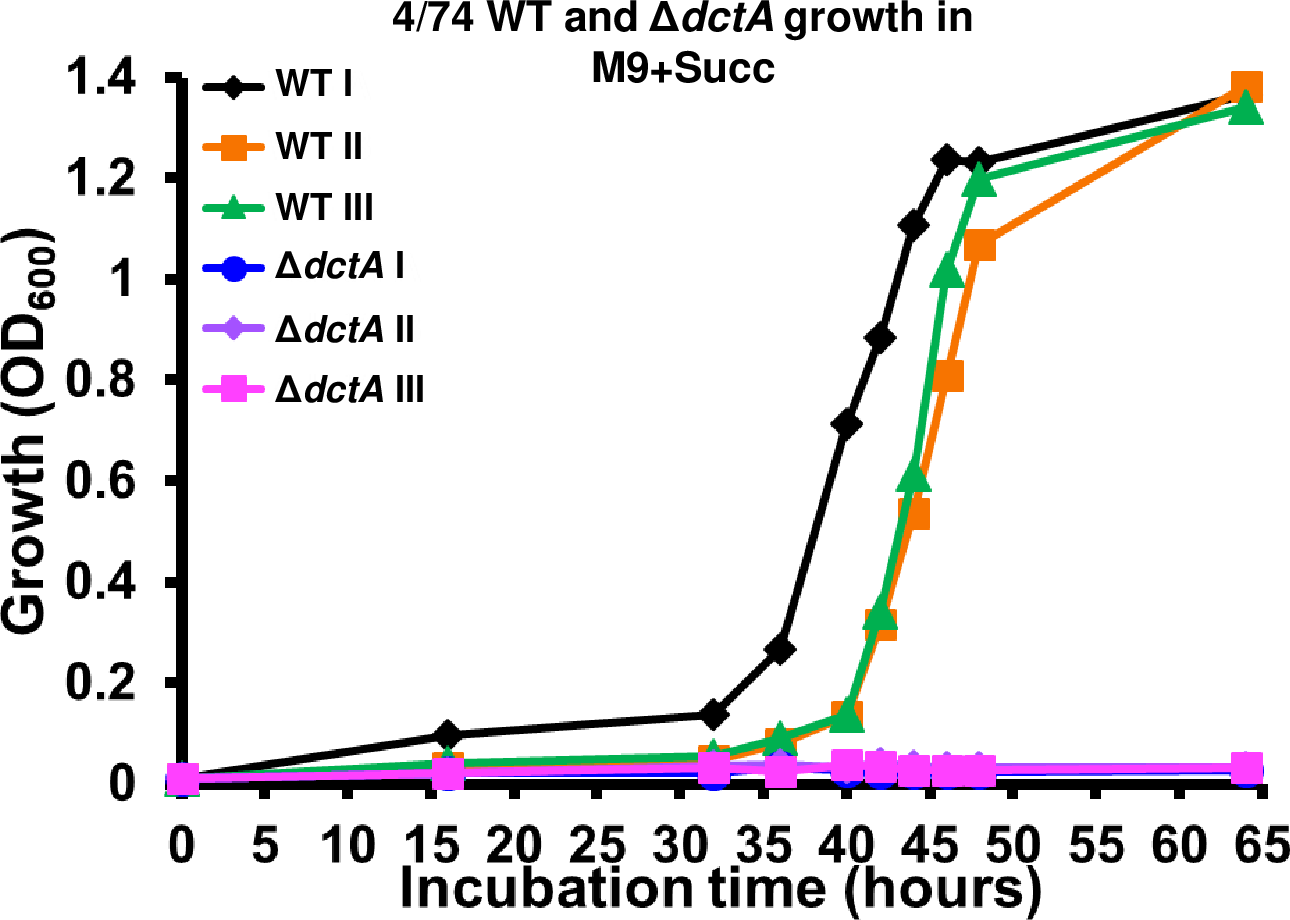

Supplement: S8 Fig — Growth of S. Typhimurium 4/74 WT displays an extended lag time, while ΔdctA does not grow at all in M9+Succ medium. The growth curves of three independent cultures (I-III) of 4/74 WT and of ΔdctA in M9+Succ are presented. bacteria were grown at 37°C with aeration in 25 ml of M9+Succ (in 250 ml conical flasks) with an initial inoculum of ~107 CFU/mL (OD600 = 0.01). (TIF) [file pgen.1011142.s010.tif]

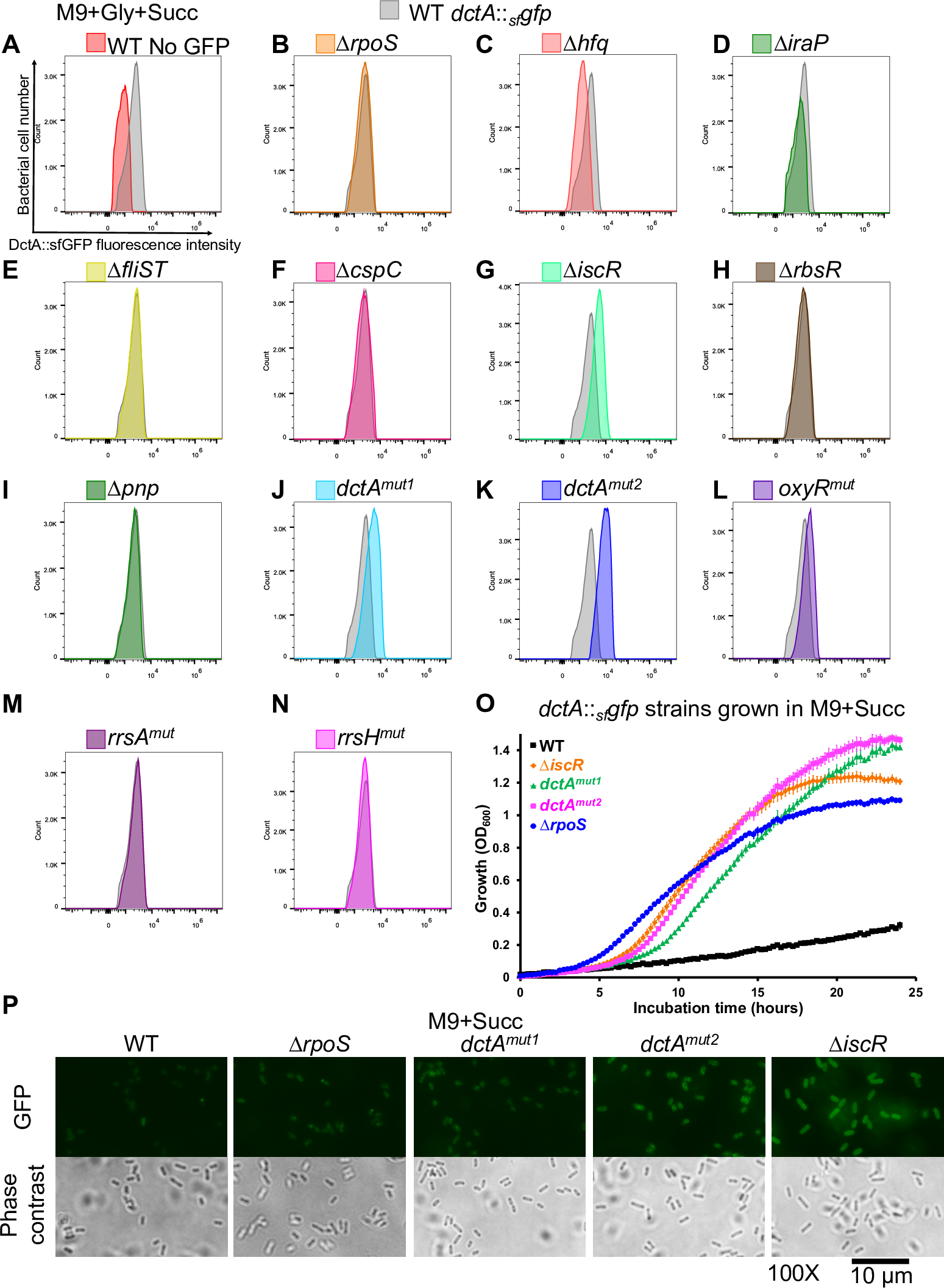

Supplement: S9 Fig — (A-N) Strains carrying the chromosomal transcriptional/translational fusion dctA::sfgfp were grown in M9+Gly+Succ minimal medium (OD600~1) and the GFP fluorescence intensity was measured with the IntelliCyt iQue Screener PLUS (Sartorius) after bacteria fixation with formaldehyde. The 4/74 WT (untagged strain) was used as a negative control (A). Each Succ+ mutants carrying dctA::sfgfp was compared with the “WT” strain carrying the same fusion (SNW296, in grey). (O) The dctA::sfgfp tagged strain ΔiscR, dctAmut1, dctAmut2 and ΔrpoS grow fast in M9+Succ in comparison with the dctA::sfgfp tagged WT strain, showing that the fusion of sfGFP to the C-term of DctA does not impede the DctA-driven uptake of succinate. (P) The same strains were grown in M9+Succ (OD600~1) and the dctA::sfgfp induction was observed by fluorescence microscopy, as specified in Methods. The dctA::sfgfp tagged Succ+ mutants used for these experiments were: ΔrpoS (SNW313), Δhfq (SNW309), ΔiraP (SNW423), ΔfliST (SNW330), ΔcspC (SNW424), ΔiscR (SNW329), ΔrbsR (SNW425), Δpnp (SNW437), dctAmut1 (SNW310), dctAmut2 (SNW316), oxyRmut (SNW426), rrsAmut (SNW374) and rrsHmut (SNW331). (TIF) [file pgen.1011142.s011.tif]

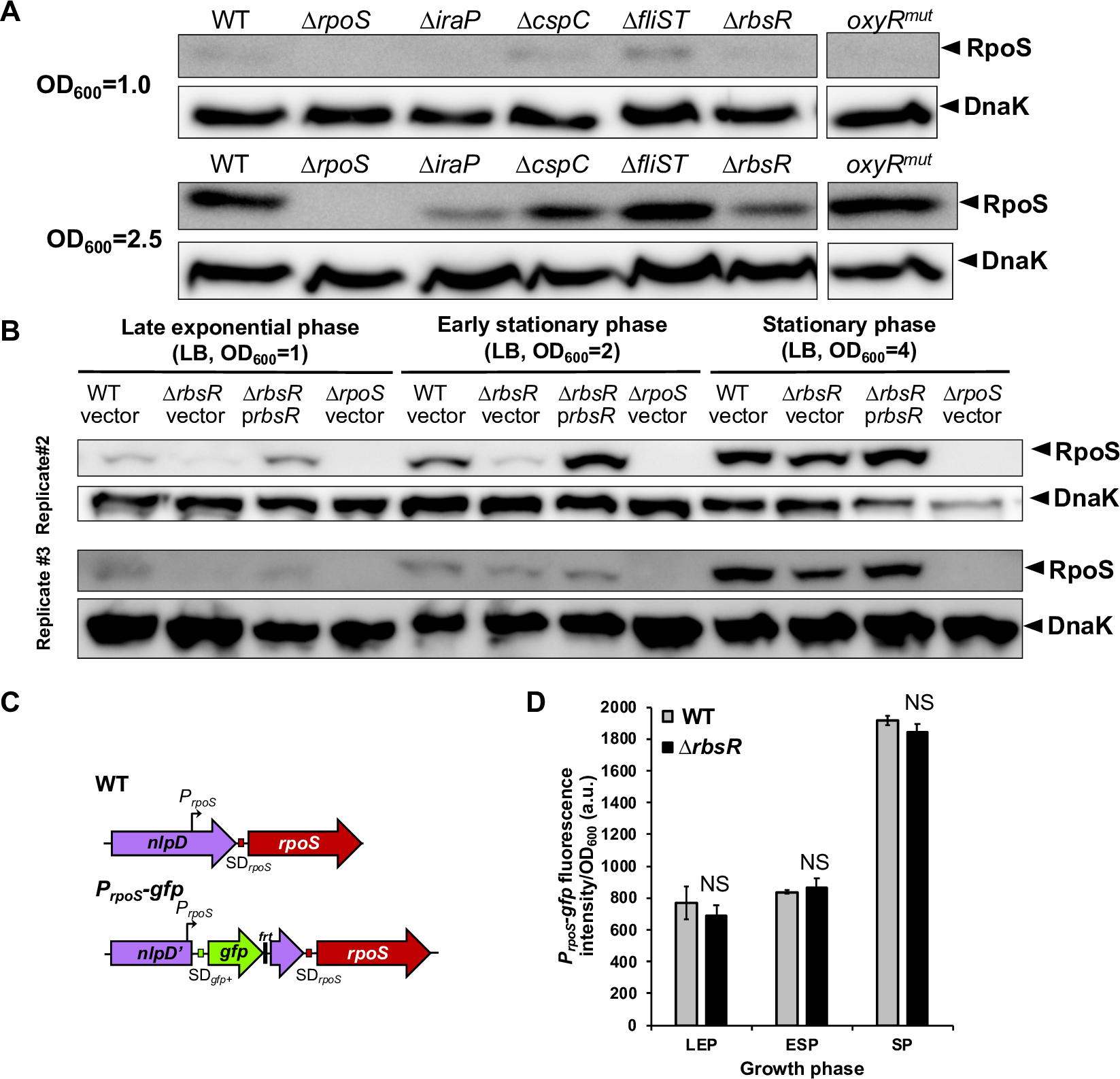

Supplement: S10 Fig — (A) Western blot detection of RpoS and DnaK (loading control) in 4/74 WT and mutants ΔrpoS (JH3674), ΔiraP (SNW188), ΔcspC (SNW292), ΔfliST (SNW288), ΔrbsR (SNW294) and oxyRmut (SNW318) grown in LB to OD600 1 and 2.5. (B) Two independent replicates of the Western blot analyses presented in Fig 7A confirmed the down-regulation of rpoS in the ΔrbsR mutant (see Fig 7A legend). (C) Schematic representation of chromosomal PrpoS-gfp transcriptional fusion. The gfp+ gene and its Shine-Dalgarno (SD) were inserted downstream of the main promoter of rpoS (PrpoS, bent arrow), interrupting the nlpD gene. The residual FLP recognition target site sequence is denoted by”frt”. The PrpoS-gfp fusion was inserted in 4/74 WT and in ΔrbsR, resulting in strain SNW367 and SNW368, respectively. (D) The PrpoS-gfp fusion activity was measured in the WT and ΔrbsR genetic background in bacteria grown in LB to late exponential phase (LEP, OD600~1), early stationary phase (ESP, OD600~2) and stationary phase (SP, OD600~4). The GFP fluorescence intensity (absolute values) were measured, as specified in Methods. The data are presented as the average of biological triplicates ± standard deviation. The difference of fluorescence intensities between the WT and the ΔrbsR strains were not significant (NS) in the three conditions tested, as defined in Methods. (TIF) [file pgen.1011142.s012.tif]

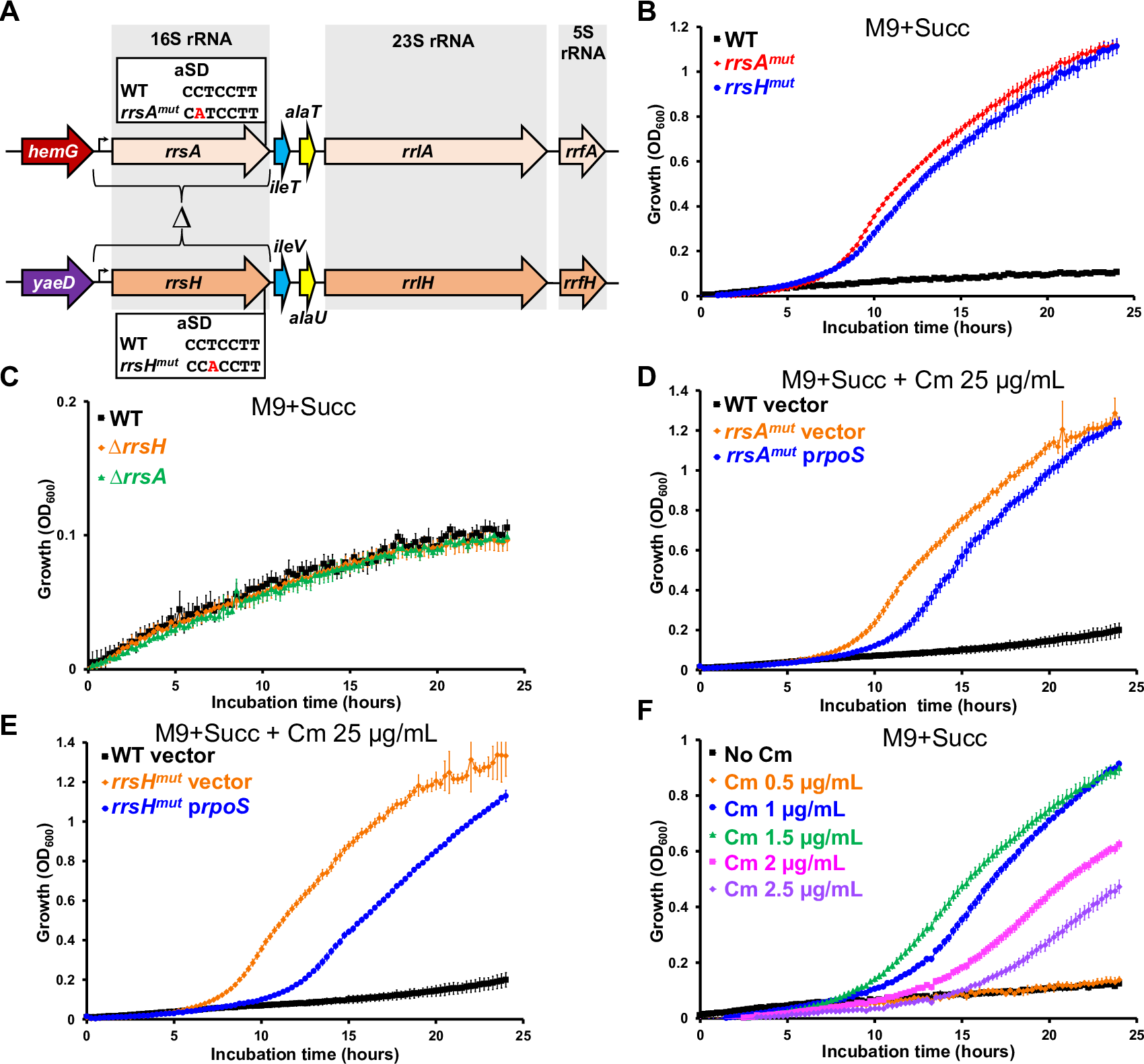

Supplement: S11 Fig — (A) Schematic representation of the Salmonella rrnA and rrnH ribosomal RNA (rRNA) operons. The 23S (rrl), 16S (rrs) and 5S (rrf) rRNAs and the ileT, ileV, alaT and alaU tRNAs are represented, according to the annotation of the corresponding loci of S. Typhimurium LT2 (Genbank AE006468.2) [130]. The bent arrows represent the ribosomal promoter. The replacement of the full rrsA and rrsH loci (promoters included) with an I-SceI-Km cassette (Methods) in strains ΔrrsA (SNW335) and ΔrrsH (SNW311) is represented by the “Δ” symbol. The SNP mutations in the anti-shine-Dalgarno (aSD) motifs of mutant rrsAmut (SNW336) and rrsHmut (SNW314) are indicated in red. (B) The aSD mutations rrsAmut and rrsHmut stimulate Salmonella growth with succinate, while the full inactivation of the rrsA and rrsH loci (strains ΔrrsA and ΔrrsH) did not affect the growth (C). The plasmid borne overexpression of rpoS has moderate effects on the growth of the rrsAmut (D) and rrsAmut (E) mutants with succinate. The 4/74 WT and the rrs mutants carried the empty plasmid (Vector, pNAW125) or the prpoS (pNAW95) plasmid. (F) Subinhibitory concentrations of chloramphenicol (Cm) stimulate Salmonella growth with succinate. All the growth curves were carried out with 6 replicates in 96-well plates with the indicated medium. (TIF) [file pgen.1011142.s013.tif]

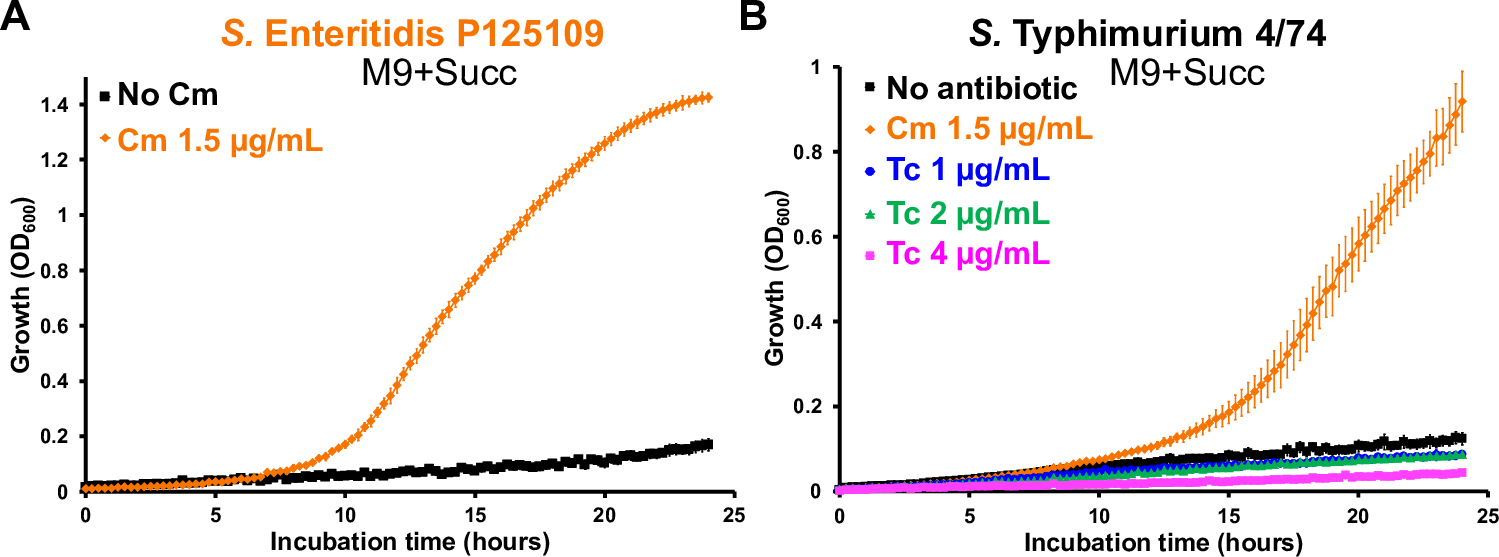

Supplement: S12 Fig — (A) Low concentration of chloramphenicol (Cm) stimulates the growth of S. Enteritidis strain P125109 and of S. Typhimurium strain 4/74 with succinate, while tetracycline (Tc) does not affect the growth profile (B). (TIF) [file pgen.1011142.s014.tif]
