# Supplementary material for: Succinate utilisation by Salmonella is inhibited by multiple regulatory systems
Source: PLoS Genet. 2024 Mar 8;20(3):e1011142. doi: 10.1371/journal.pgen.1011142 (PMC10965054; doi:10.1371/journal.pgen.1011142)
Supplement: Attachment — Submitted filename: 133374_1_rebuttal_2814590_s23yxx.pdf [file pgen.1011142.s017.pdf]

Full Revision

Manuscript number: RC-2023-01830
Corresponding author(s): Nicolas, Wenner

[Please use this template only if the submitted manuscript should be considered by the affiliate journal as a full revision in response to the points raised by the reviewers.

If you wish to submit a preliminary revision with a revision plan, please use our "Revision Plan" template. ***It is important to use the appropriate template to clearly inform the editors of your intentions.****]*

1. General Statements [optional]

We are grateful to the Reviewers for their insightful thoughts and suggestions for improving the manuscript for publication. We have addressed all Reviewers' comments, and detailed responses have been provided below (in blue font). We have uploaded a revised manuscript version, and have made a few small improvements to the text to improve readability. Line and figures numbers refer to the revised version of the manuscript.

2. Point-by-point description of the revisions

'Reviewer #1 (Evidence, reproducibility and clarity (Required)):

In this study, Wenner et al. used various in vitro methods, including transposon mutagenesis, screening of known regulatory proteins and isolation of spontaneous mutants to discover 11 mutations in genes that promote bacterial growth under succinate-mediated inhibition. Through additional experiments, the manuscript provides evidence for factors that underlie several layers of succinate regulation. These layers include sRNAs, OxyRS, succinate transport antibiotics and rRNA. The study then characterized the molecular mechanisms regulating succinate utilization by these mutations, revealing a RpoS-independent mechanism for succinate uptake via the dctA transporter and mechanisms for RpoS regulation.

Overall, the manuscript is very unfocused and uneven in the level of details of each of these factors and could be much more compelling if more focus was given to several factors and providing more mechanistic insight of these factors.

We thank Reviewer #1 for the constructive criticism and suggestions. We do recognize the limitations of our study, clearly more work is required to unravel the complex phenomenon of the

inhibition of succinate utilisation by *Salmonella*. We welcome Reviewer #1's suggestions to shorten the manuscript, which has allowed us to focus the paper on our key findings.

Major comments
1. The authors discuss virulence-mediated succinate but disregard some important features of succinate utilization, only referring to dctA and disregarding the overlap with other C4-dicaroxy transporters (Spiga, wolf, PMID). Furthermore, the study found that a mutation in the IscR binding site on the DctA promoter region reversed the effects of succinate-dependent growth inhibition generated under aerobic conditions but other succinate transporters are expressed under different physiological conditions (Janausch et al. 2002, Spiga et al. 2017).
Does the IscR binding site motif can be found in promoters of other succinate transporters? Analysis of □IscR in aerobic/ anaerobic conditions can be useful. Do mutations in IcsR lead to increased expression of other succinate transporters in aerobic or anaerobic conditions?

The Reviewer's question of the regulatory role of IscR on anaerobic C_4-dicarboxylate transporters is particularly relevant in the context of the role of succinate catabolism in pathogenesis and could be studied in a follow-up investigation. However, further analysis of the influence of mutations that modulate the expression or activity of IscR are beyond the scope of our study. Here, we have focused on succinate utilisation under *in vitro,* aerobic conditions: under these conditions, growth upon succinate is robustly repressed, allowing the selection of $Succ^+$ mutants. To emphasise that our study was done under aerobic conditions, we have rephrased the Introduction (line 93).

2. Transposon screen - There is no comprehensive description of the results and it is not clear why mutations found in the evolution experiments or regulatory proteins that were shown to allow bacteria growth under succinate treatment were not detected in the transposon screening?

Different selection protocols were used to isolate the $Succ^+$ mutants and the experimental approaches are detailed in the Methods and in the strain list for each mutant (Supplementary_Resource_Table S1). Selection was performed in liquid M9+Succ for Tn*5* mutagenesis (in the $rpoS^{2X}$ background), or in solid and liquid M9+Succ media for the spontaneous mutants (the mutations are all listed and detailed in Table 1).
Therefore, the different selection conditions and the presence of an extra *rpoS* copy may have favored certain mutants, especially when the pools of Tn*5* mutants were grown with succinate together (mutants in competition).
We recognize that our experimental approach had limitations, and that a Tn-seq methodology would have been more comprehensive. However, the robustness of the phenotypes of the mutants (all re-constructed and complemented, when possible) demonstrated that the genes of interest had direct impact upon the control of succinate metabolism with novel implications for the field.

3. Figure 4: The authors claim: "that the fast growth of the Δhfq and Δpnp strains reflected both the dysregulation of the sRNA-mediated repression of sdh and the activation of rpoS translation". However, they provide no evidence for SDH regulation. The experiment is correlative, the activity

of pnp regulating rpoS was done with overexpression without the proper controls. The authors should look at rpoS expression in Δpnp. It does not seem reasonable that transcription of SDH mRNA can explain lack of succinate utilization. What about the SDH protein? is it at all changed? The authors claim "none of the sRNA mutants tested displayed the same fast-growing pattern of the Δhfq mutant" but they action can involve completely different mechanisms, that the authors do not study. This part does not seem to contribute any novel information on Δhfq and Δpnp on Succ+ with the sRNAs seem not to provide any clear mechanism. The authors should consider removing this part or moving to supplementary.

We appreciate this comment, and agree that this section does not provide critical novel insight. However, our findings provide valuable data concerning the role that Hfq, PNPase and sRNAs play in succinate utilisation. Therefore, we have briefly mentioned the role of Hfq, PNPase and sRNAs in the main text (Lines 333-338) and moved the original Figure 4 to the Supplementary (Figure S5), with a supplementary text section (Supplementary Text T1).

4. If OxyS, an Hfq-binding sRNA, is related to Succ+ in Δhfq, then why all the other sRNAs are relevant? This is not clear. The authors could have focused here on the oxyS instead of other sRNAs. "The same plasmid did not stimulate the growth of the ΔoxyR strain indicating that a functional OxyR is required for growth in M9+Succ (Fig 5D)" - is it because of other targets of OxyR?

The reviewer's interpretation is correct. To clarify this point, we have rephased the sentence (Lines 274-276) to "The same plasmid did not stimulate the growth of the Δ*oxyR* strain indicating that other OxyR-dependent genes are required to grow under this condition"

It seems that an RNA-seq analysis in the conditions of succinate growth with OxyRmut vs. WT could hint towards this.

Indeed, it would be very interesting to compare the transcriptomic landscape of the WT and of the *oxyR*^{mut} mutant and other Succ^{+} mutants in succinate minimal medium. However, the lack of growth of *S.* Typhimurium WT in M9+Succ, would make these experiments unlikely to succeed.

"We previously showed that Hfq inactivation boosted succinate utilization (Fig 4A), but in the oxyRmut genetic background the same Hfq inactivation dramatically reduced growth and extended the duration of lag time in M9+Succ (Fig 5 E)"

The reviewer is correct, we had hypothesised that Hfq is necessary to stimulate succinate utilisation by OxyS. Therefore, we have rephrased to: "We previously showed that Hfq inactivation boosted succinate utilisation, but in the *oxyR*^{mut} genetic background the same Hfq inactivation dramatically reduced growth and extended the duration of lag time in M9+Succ (Fig 4E). Collectively, our findings show that the OxyS sRNA orchestrates the de-inhibition of succinate utilisation in concert with Hfq" (Lines 278-281)

- this seems like an interesting finding, but the authors don't offer any follow-up? Is it related to oxyS activity?

The role of Hfq on succinate utilisation appeared to be dual, we have added a sentence to this effect (Lines 335-338).

5. Figure 6: "OxyS acts as an indirect repressor of RpoS expression, probably via the titration of Hfq". the yobF::sfgfp activity was significantly lower in the oxyRmut strain (~2-fold repression), confirming that OxyS represses the expression of the yobF cspC operon in Salmonella - can the authors show this directly with oxyS in succinate?

Because *Salmonella* WT and Δ*oxyS* strains do not grow in succinate media (M9+Succ), we had to investigate the regulation of *yobF-cspC* operon with a translational gene fusion in non-selective LB media.

Why use OxyRmut here? This is indirect.

In Figure 5C we first used the *oxyR*^{mut} Succ^{+} strain to demonstrate that this mutation leads to the repression of *yobF-cspC*. In Figure 6F, we used the *oxyR*^{mut} allele to allow a constitutive expression of *oxyS* WT or *oxyS*^{GG} : allele *oxyS*^{GG} was introduced into the chromosome and relies on an active OxyR to be transcribed. The direct role of OxyS is demonstrated in Figure 5 E &F.

The authors already show that OxyRmut does not act solely via Oxys...can the authors directly show RpoS and SDH levels by qRT-PCR in ΔcspC? Again - the appropriate control for RpoS overexpression in the WT was not done (Fig. 6G). Furthermore, expression analysis of the sdhCDAB operon over the background of the oxyR mutant will confirm the author suggestion for the mechanism by which the OxyS-driven inhibition of CspC expression impacts upon the catabolism of succinate.

The reviewer's comments are valid, more work is required to understand how OxyS stimulates succinate utilisation *via* the repression of *cspC*. The fact that *Salmonella* WT does not grow with succinate as a sole carbon source makes such comparisons technically challenging. Yes, the repressive role of CspC remains enigmatic. However, RNA-seq data following growth in LB media have already been provided by others, suggesting that CspEC may repress TCA cycle genes in *Salmonella* (PMID: 28611217), consistent with the repression of succinate catabolism by CspC.
The fact that the plasmid-borne overexpression of *rpoS* completely represses growth upon succinate in the Δ*rpoS* background (Figure S3 B) validated the usage of the p*rpoS* plasmid in other genetic backgrounds, in order to reveal whether the other Succ^{+} mutations were stimulating succinate utilisation *via rpoS* repression or not. Because WT *Salmonella* does not grow in M9+Succ, presenting the growth curve of the WT strain carrying the p*rpoS* plasmid would not be informative here, and would make the figure overly complex.

6. Figure 7: the authors check growth in M9+succ in the absence of DctA - but the experiment duration should be carried out for longer, as previous experiments with WT (intact dctA in Fig. 2A) and check if in the absence of dctA there are mutations that allow succinate growth.

We agree with the reviewer's comment and we have performed a new growth curve (over 65 hours) of the Δ*dctA* strain to clarify that DctA is the only succinate transporter involved in *Salmonella* growth under our experimental conditions (Figure S8).

It seems that the results here contradict some of the previous - if succinate uptake through dctA is intact then there is no repression of SDH? rpoS? In figure 7E - is this difference only through dctA activity?

The reviewer is raising an important point and it is possible that the de-repression of succinate uptake *via* DctA could impact upon the expression of the succinate catabolic genes and more work is required to understand this phenomenon. We have discussed this possibility in the main text (Lines 424-432) and in Figure 8C.

It seems that □icsR is not repressing dctA expression to WT levels - are there other factors? Can the authors show that dctA repression by IscR is direct?

We agree with the reviewer, we have not shown that IscR represses *dctA* directly. Electrophoretic mobility shift assays could be performed to prove that IscR interacts with the *dctA* promoter region, but this would be beyond the scope of the paper. We have clearly stated in the discussion that indirect effects of *iscR* on *dctA* expression cannot be ruled out (Lines 419-422).

7. Figure 9 is very descriptive and does not provide any evidence to support the authors hypothesis. The authors should either provide more substantial evidence connecting ribosomal RNA levels and succinate utilization and similarly Cm concentrations or either remove this part or move it to the supplementary.

We agree that the data do not conclusively support the hypothesis, but we believe that the impact of anti-SD mutation and chloramphenicol on *Salmonella* carbon metabolism are valuable observations for the community. Therefore, we have moved the data to supplementary Figures S11 and S12 in the revised version, with a supplementary text section (Supplementary Text T2). We also removed this aspect from the model Figure (Figure 8) and only mentioned the phenomenon briefly in the main text, Lines 482-485.

8. Can any of the mutations characterized in this work be found in the genome of Newport or LT2 strains that can grow with succinate as a sole carbon source? (Fig 1)

Very good questions. Yes, *S.* Typhimurium strain LT2 has an altered *rpoS* allele that attenuates virulence of the strain in the murine infection model (PMID: 8975913) and promotes growth with

succinate (PMID: 33593945). We have added a sentence and cited the reference at Lines 129-131.

To address the *S.* Newport question, we performed an analysis of the genome of the *S.* Newport strain LSS-48, and did not identify any mutations in regulatory or catabolic genes that could explain the faster growth on M9+succinate. However, in comparison with fast-growing enteric bacteria (*i.e. E. coli* MG1655) or Succ$^+$ *S.* Typhimurium mutants, *S.* Newport LSS-48 grows much slower on succinate and has an intermediate growth phenotype. It remains unclear why S. Newport does grow better than other serovars.

9. Although the author suggested that regulation of succinate uptake is critical for Salmonella colonization and virulence in various metabolic conditions, the study lacks sufficient evidence to support these claims and further research is necessary to establish these statements.

We agree that our findings are not directly linked to *Salmonella* host colonisation or virulence. However, we do believe that our study will contribute to a better understanding of *Salmonella* metabolic control, in the context of pathogenesis. To address Reviewer #3's comment, we have moderated our claims about the likely impact of our findings on the understanding of *Salmonella* pathogenesis in the Perspective section.

Minor comments
1. Table summarizing the growth curves lag phase of the different mutants might help in the data interpretation.

We appreciate the Reviewer's suggestion and have prepared a supplementary figure (Figure S4) indicating the average lag time of the Succ$^+$ mutants and of the complemented mutants.

2. In lines 245-248 the author describes the eleven novel Succ+ mutations however in this gene list only ten gene names are mentioned. DctA is missing from this list.

We appreciate the Reviewer's comment and we have modified the sentences in the revised manuscript (Line 244).

** Referees cross-commenting**
I agree with both reviewer that there is a large amount of data in the paper, and willing to accept their point that asking for further experiments would exceed the scope of the paper. In that case, the authors should address the mechanistic options in the discussion
Reviewer #1 (Significance (Required)):
In this work, Wenner et al. characterized the molecular mechanisms regulating Salmonella growth inhibition when succinate is the sole carbon source in the culture. This work revealed new layer of regulations for rpoS activation, the sigma factor previously characterized to control this growth inhibition mechanism. In addition, this work revealed novel RpoS-independent mechanisms for

succinate utilization and highlighted the crucial role of succinate processing in Salmonella physiology.

Reviewer #2 (Evidence, reproducibility and clarity (Required)):

In the manuscript titled "Salmonella succinate utilisation is inhibited by multiple regulatory systems", Wenner et al., explored how Salmonella regulates the utilization of succinate, an important carbon source for Salmonella gut colonization as well as a molecule that regulates intracellular adaptation in the SCV. As Salmonella exhibits a slow growth rate when succinate is provided as the sole carbon source, the authors explored the underlying genetic regulation by isolating fast-growing mutants (Succ+) using an experimental evolution approach. By combining the screen for mutants lacking key regulatory proteins, an elegantly designed Tn5 transposon mutagenesis, and selection of spontaneous Succ+ mutants, the authored identified a library of mutations that led to the Succ+ phenotype. Using classical bacterial genetics, Wenner et al characterized how Hfq, PNPase and cognate sRNA inhibit succinate utilization. They went on to show, clearly and convincingly, that IscR inhibits growth upon succinate by repressing DctA expression, and succinate utilization can also be repressed by RbsR and FliST via RpoS. Lastly, they provided evidence supporting that anti-Shine-Dalgarno mutations and low concentrations of chloramphenicol can boost succinate utilization. Overall, this paper is well written, and the experiments were rigorously designed and executed. This is a beautiful example of deciphering complex regulatory nodes in the succinate utilization using elegant genetics approaches. Very nicely done!

We thank the Reviewer #2 for the very positive evaluation of our work and the constructive comments.

Minor issues:
1. While rpoS2X strain is an clever way to avoid the selection of Succ+ rpoS mutants, it is unclear why "identified an iraP::Tn5 mutant was an effective validation of the use of the rpoS2X genetic background". IraP stabilizes Rpos, and this mutant could have been selected in the wild-type background (rpoS1X).

The reviewer’s comment is helpful, we have removed this sentence from the revised manuscript.

2. The description between line 356-357 is confusing as it reads like the author constructed a "oxyRmut oxySGG pPL-OxySGG" strain, while the experiments that followed actually used a " ∆oxyS, yobF::sfgf, pPL-OxySGG" strain.

We have modified these sentences in the revised manuscript (Lines 303-308).

3. An alternative explanation for the Succi+ phenotype in aSD mutant and bacteria treated with low Cm is the reduced translation fidelity, which leads to selectively degradation of inhibitors of succinate utilization.

We thank Reviewer #2 for the suggestion. This phenomenon is really enigmatic and as previously discussed in Reviewer #1's section, we have now moved Figure 9 to supplementary data. Further discussion of how the aSD mutations and chloramphenicol can affect *Salmonella* succinate metabolism would require a lot more experimental data.

** Referees cross-commenting**

Most of the comments from Reviewer 1 are valid but excessive. Most of the experiments presented in this paper were rigorously controlled and executed. While some parts of the paper could be more mechanistic but they could also leave room for future studies. Also, some of the points raised, the 1st major concern, for example, may have exceeded the scope of the paper.

We agree. We have performed a new experiment (Figure S8) to address Reviewer #1's comments.

Reviewer #2 (Significance (Required)):

Overall, this paper is well written, and the experiments were rigorously designed and executed. This is a beautiful example of deciphering complex regulatory nodes in the succinate utilization using elegant genetics approaches.

We appreciate Reviewer #2's feedback that the quality of the text and our experiments was viewed so highly.

Reviewer #3 (Evidence, reproducibility and clarity (Required)):

In this work, Wenner and colleagues use experimental evolution to define a range of spontaneous mutations in Salmonella that allow it to overcome its aversion to using succinate as a carbon source in vitro.

This work cites the literature extensively and the scholarship is very very good. I appreciate the effort they put into the manuscript, which made it easy to read. Quite a relief to get a paper in this good of shape compared to most.

We appreciate Reviewer #3's positive comments on our work and the constructive suggestions.

Shortcomings - although I don't think they are necessary for *this* paper to be published include:
- not defining what could be 'bad' about eating succinate in the wrong place. The fact that succinate import is a problem (dctA is what is being regulated ant its a transporter) suggests one of the following: (1) excess succinate would block the utilization of fumarate by fumarate reductase, (2) succinate is a powerful buffer and, if protonated, would acidify the cytoplasm of Salmonella if it were brought in - note that there is a lot of work on RpoS controlling cytoplasmic acidification, (3) a drop in succinate (because Salmonella eats it) would allow more flux by macrophages or the microbiota in a bad way...maybe the Salmonella 'wants' macrophages to have lots of succinate *because* its pro-inflammatory (and therefore more tetrathionate for its friends...etc), (4) it could be the transporter that also bring in antimicrobial itaconate?...so the succinate phenotype is a red herring and really this is about preventing taconite from getting into the cell?

We thank the reviewer for all these suggestions and for highlighting the reasons why the avoidance of *Salmonella* utilising succinate is a key point. We have emphasized this key question to conclude our manuscript (Lines 500-501). Whilst all the hypotheses are valid, we believe that further speculation should not be added to the "Perspective" section.

- no proof that any of this is relevant in infection except citing old papers. Again - this work is already VERY expansive and we could propose experiments until the end of time. Next paper should take the dctA and other mutations and put them into mice to see if they fail in either germ free mice (no microbial produced succinate around) or in systemic infections.

The reviewer's comment is welcomed. As discussed in our response to Reviewer #1, we have scaled back our discussion of the impact of our findings for the understanding of *Salmonella* pathogenesis.

Most of the mutations they find are 'regulatory' and the only proximal effector of succinate utilization seems to be dctA...suggesting that dctA expression is the 'rate limiting' or 'blocked' step that decides whether succinate is being used or not.

We agree that *dctA* regulation is a central element of the story. As discussed in Reviewer #1 comments, it is not clear how de-repression of *dctA* leads to the increased catabolism of succinate in the presence of RpoS (particularly because RpoS represses several succinate catabolic genes, PMID: 24810289 and PMID: 25578965). We also discovered other Succ$^+$ mutants that did not affect DctA expression but stimulated growth on succinate as a sole carbon source. Consequently, it is uncertain whether the uptake of succinate is really the limiting factor. We have added sentences about this paradox, Lines 424-432.

The data is extensive and generally well controlled. Where appropriate they either complement mutations or reconstruct them denovo. The findings of the various genes range in novelty but many are new.

** Referees cross-commenting**

I agree that the work was valid and well controlled. The 'story' was a bit disjointed at times primarily because the range of mutations identified were diverse and pleiotropic. Given the large amount of data already in the paper and the nature of the mutations identified I worry about embarking on an endless cycle of new experiments. I think it's at a publishable stopping point.

In response to Reviewer #3 & #1's comments, we have now improved the flow of the manuscript.

Reviewer #3 (Significance (Required)):

This seemingly mundane phenotype (Salmonella 'choosing' to not use succinate even though it's perfectly capable of doing so) has been known for years but only recently has its potential relevance become more clear in the context of infection and microbiota metabolism.

The authors propose that succinate utilization is to be used at the right time and right place.

I sympathize with the authors that they keep hitting very pleiotropic regulators (RpoS has ten million upstream inputs and outputs. The ribosome? How is that going to be figured out in one or two simple experiments?). My money is on figuring out exactly how dctA is regulated and whether there's differences in the dctA regulation between E. coli and Klebsiella/Salmonella.

So I think the work is extensive and generally well done. I think the paper will be well cited...and I think it's importance will grow over time and it will continue to be relevant years from now. I can't say that about most work in the field.

We agree with Reviewer #3's assessment that other scientists in the *Salmonella* field are likely to cite our paper, and to perform experiments that will build on our findings in the future.
